# Wide QRS Complex Tachycardia in a Patient With Acute Myocarditis Temporarily Protected With a Wearable Cardioverter-Defibrillator

**DOI:** 10.7759/cureus.112342

**Published:** 2026-07-09

**Authors:** Adam Wojtaszczyk, Jerzy K Wranicz, Iwona Cygankiewicz, Krzysztof Kaczmarek

**Affiliations:** 1 Department of Electrocardiology, Medical University of Lodz, Lodz, POL

**Keywords:** cardiac sudden death, heart failure, myocarditis, wearable cardioverter-defibrillator, wide qrs tachycardia

## Abstract

A wearable cardioverter-defibrillator (WCD) provides noninvasive and temporary protection against sudden cardiac death (SCD) in patients at transient risk of ventricular arrhythmias, including myocarditis. We report the case of a 68-year-old man with recurrent wide QRS complex tachycardia (WCT) and myocarditis confirmed by cardiac magnetic resonance imaging, who was discharged with a WCD following in-hospital stabilization. During the one-month follow-up, the device detected 200 WCT episodes, lasting up to 50 seconds and reaching rates of 150 beats per minute, despite clinical improvement and recovery of left ventricular ejection fraction (from 47% to 52%). An electrophysiological study revealed orthodromic atrioventricular reentrant tachycardia mediated by a retrogradely conducting accessory pathway (AP), reproducing the WCT morphology recorded by the WCD. Radiofrequency ablation of AP successfully eliminated the arrhythmia. This case highlights the diagnostic value of prolonged electrocardiographic monitoring with WCD beyond protection from malignant arrhythmia, enabling timely intervention and avoidance of unnecessary implantable cardioverter-defibrillator implantation.

## Introduction

A wearable cardioverter-defibrillator (WCD) is a non-invasive external device designed to prevent sudden cardiac death (SCD) due to ventricular arrhythmias (VA). The system consists of a wearable vest equipped with diagnostic and therapeutic electrodes and a central unit with batteries that continuously monitor and analyse a patient's heart rhythm. This medical device has been developed for patients with a transient, potentially reversible increased risk of SCD or for those with temporary contraindications to implantable cardioverter-defibrillator (ICD) implantation [1,2]. Current clinical practice guidelines identify several scenarios, including acute myocarditis (AM) and the early post-myocardial infarction period, in which the use of a WCD may be considered appropriate [3]. AM is associated with an increased risk of VA, especially among high-risk patients, including those with impaired left ventricular ejection fraction (LVEF) and those presenting with syncope [4,5]. Beyond its therapeutic function, the WCD provides continuous remote monitoring, yielding clinically relevant information on patient status [6,7]. In addition to electrocardiographic data, the device records heart rate trends, physical activity (step count), and body position, which may facilitate the early detection of clinical deterioration [8,9].

## Case presentation

**Figure 1:**
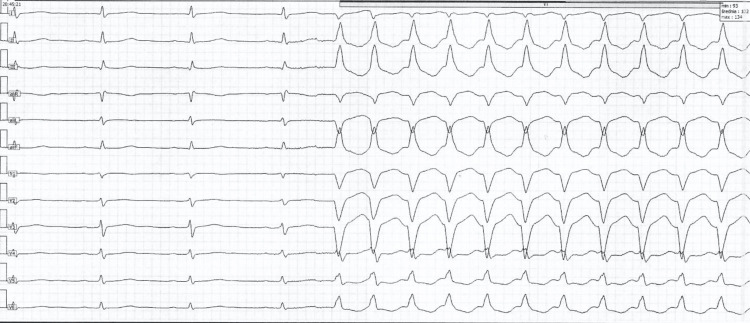
Initiation of the wide QRS tachycardia recorded on 12-lead Holter monitoring.

A 68-year-old man was transferred to the Department of Electrocardiology from a tertiary cardiology centre because of recurrent, self-terminating episodes of sustained and non-sustained, hemodynamically tolerated wide QRS complex tachycardia (WCT; up to 150 bpm) documented on Holter monitoring (Figure 1).

The patient's medical history was notable for hypertension. One month before admission, he developed new-onset heart failure symptoms, including dyspnea, reduced exercise tolerance (New York Heart Association class III), and dizziness, accompanied by dynamic elevations in cardiac troponin levels (57 ng/L; normal <14 ng/L). Coronary angiography performed at the referring center revealed only non-significant coronary artery stenoses. Following the procedure, the patient experienced an episode of ventricular fibrillation (VF) requiring external defibrillation. During subsequent observation, several episodes of non-sustained WCT and frequent premature ventricular complexes were documented. Prior to this episode, the patient had not reported any palpitations. Analysis of the WCT electrocardiogram yielded 1 point on the VT score, based on an R-wave peak time in lead II >50 ms [10].

Based on the clinical presentation and electrocardiographic findings, ventricular arrhythmia was initially suspected. Transthoracic echocardiography demonstrated global left ventricular hypokinesia with mildly reduced LVEF (47%) and moderate mitral regurgitation. Cardiac magnetic resonance (CMR) imaging revealed further impairment of LVEF (42%) and intramural and subepicardial areas of late gadolinium enhancement within the interventricular septum and the inferior and lateral walls of the left ventricle, consistent with active myocarditis.

Beta-blocker therapy was initiated at a low dose because of symptomatic bradycardia. Amiodarone was discontinued due to QTc prolongation. No further arrhythmias were observed during the subsequent 12-day hospitalization.

Given the moderately reduced LVEF, the episode of VF, recurrent WCT episodes, and CMR features consistent with active myocarditis, the patient was discharged with a wearable cardioverter-defibrillator (WCD; LifeVest®, Zoll Medical Corporation, Chelmsford, MA) for temporary protection against sudden cardiac death.

**Figure 2:**
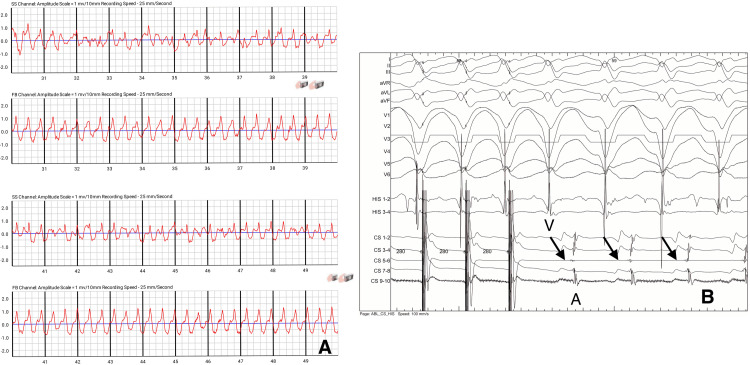
Registration of an arrhythmic episode from a wearable cardioverter-defibrillator (A). Induction of orthodromic atrioventricular re-entrant tachycardia during an electrophysiological study. The atrioventricular ratio was 1:1. The earliest atrial activation was recorded on coronary sinus electrodes 5-6, corresponding to the posteroseptal region. V: ventricular activation; A: atrial activation (B). CS: decapolar catheter positioned in the coronary sinus; HIS: quadripolar catheter positioned in the right ventricle.

One month later, the patient was readmitted after the WCD detected approximately 200 episodes of WCT lasting up to 50 seconds and reaching rates of 150 bpm (Figure 2A). All episodes were mildly symptomatic. Potential therapies were aborted by the patient using the response buttons on the device, and therefore no shocks were delivered. Cardiac troponin levels on readmission were within the normal range, and repeat CMR demonstrated improvement in LVEF (52%) together with resolution of the active inflammatory process (Table 1).

**Table 1:** Comparison of clinical parameters at baseline and after one-month follow-up. NYHA: New York Heart Association; LVEF: left ventricular ejection fraction; CMR: cardiac magnetic resonance; WCT: wide QRS complex tachycardia; WCD: wearable cardioverter-defibrillator; EPS: electrophysiological study; oAVRT: orthodromic atrioventricular reentrant tachycardia; ICD: implantable cardioverter-defibrillator

Clinical parameter	Baseline	1-month follow-up
NYHA class	III	I
Troponin	57 ng/L	Normal
LVEF (CMR)	42%	52%
Active Myocarditis features (CMR)	Present	Resolved
WCT episodes	Recurrent during index hospitalization	~200 episodes (up to 50 s, 150 bpm)
WCD therapy	Initiated	No appropriate shocks; therapies aborted by patient
EPS	Not performed	oAVRT with aberrant conduction diagnosed
Catheter ablation	No	Successful
ICD indication	Considered	Avoided after diagnosis and LVEF recovery

An invasive electrophysiological study (EPS) was performed for further risk stratification. The study revealed a retrogradely conducting accessory pathway located in the proximal coronary sinus (left posteroseptal region), with an effective refractory period of 240 ms. Programmed atrial stimulation induced orthodromic atrioventricular reentrant tachycardia (oAVRT), with intermittent aberrant conduction, reproducing the morphology of the WCT previously recorded on ECG (Figure 2B). Radiofrequency ablation was performed using a 4-mm irrigated-tip catheter with a power setting of 30 W at the ostium of the coronary sinus, resulting in the successful elimination of the accessory pathway and rendering the arrhythmia non-inducible. Post-ablation EPS confirmed the absence of inducible arrhythmias, and WCD therapy was subsequently discontinued.

At 12-month follow-up, the patient remained free of arrhythmia based on clinical assessment and Holter ECG monitoring.

## Discussion

The WCD represents a therapeutic option for patients with a transiently increased risk of arrhythmic death, including those with AM [4]. It may also be considered in other high-risk populations, such as patients in the early post-myocardial infarction period (<40 days) with LVEF <35% [3]. Although the primary intention-to-treat analysis of the Vest Prevention of Early Sudden Death (VEST) trial did not demonstrate a significant reduction in SCD, subsequent per-protocol analyses revealed a significant benefit. This discrepancy was largely attributed to suboptimal adherence, with most deaths occurring when patients were not wearing WCD [1,11].

VAs are common in patients with AM and are associated with a worse prognosis in this population [12]. Although most episodes occur during the acute phase of the disease, the risk of VA may persist during long-term follow-up [13]. In the present case, the origin of the WCT was initially uncertain. A VT score of only one point was insufficient to establish a diagnosis of VT [10]. Nevertheless, the possibility of a ventricular origin, combined with the previous episode of VF following coronary angiography and the presence of other high-risk clinical features, supported the decision to prescribe a WCD. Given the inconclusive electrocardiographic findings, an EPS was performed to determine the mechanism of the arrhythmia. Grbović et al. demonstrated that this strategy may also be valuable in patients with coronary artery disease presenting with WCT [14]. This stepwise diagnostic approach ultimately excluded VT. Together with the recovery of LVEF, the patient was no longer considered to be at high risk of SCD, allowing unnecessary ICD implantation to be avoided.

The present case also highlights the broader clinical value of the WCD beyond temporary protection against SCD. Incorporating the WCD into routine clinical practice not only provides effective protection during the transient high-risk phase but also creates a therapeutic window for myocardial recovery and more accurate risk stratification. Recent data from the Polish WCD registry demonstrated that permanent ICD implantation was ultimately avoided in approximately 50% of patients managed with a WCD [2,15].

In the present case, remote monitoring through the WCD provided valuable additional information regarding the patient's clinical course. The unexpectedly high burden of recurrent WCT episodes prompted earlier reassessment and hospital admission despite clinical improvement, leading to timely electrophysiological evaluation and definitive catheter ablation. This case illustrates that the clinical utility of the WCD extends beyond arrhythmia detection and therapy. Wearable medical devices have assumed an increasingly important role in the diagnosis and management of arrhythmias in recent years [16]. Continuous remote monitoring provides clinically relevant information, including electrocardiographic and physiological data, facilitating early recognition of clinically significant changes and enabling prompt therapeutic intervention [6,7].

## Conclusions

This case demonstrates that the WCD may provide clinical value beyond temporary protection against SCD in patients with AM. Continuous WCD monitoring facilitated early recognition of recurrent WCT, prompting timely electrophysiological evaluation and establishing the diagnosis of oAVRT with aberrant conduction. Accurate arrhythmia identification enabled successful catheter ablation and avoided unnecessary ICD implantation. This case highlights the complementary protective and diagnostic role of the WCD and emphasizes the importance of a stepwise diagnostic approach in patients with ambiguous WCT.

## References

[1] Olgin JE, Pletcher MJ, Vittinghoff E (2018). Wearable cardioverter-defibrillator after myocardial infarction. N Engl J Med.

[2] Wojtaszczyk A, Kaczmarek K, Urbanek I, Cygankiewicz I, Wranicz JK (2024). Wearable cardioverter-defibrillator in daily clinical practice: a single-centre experience. Kardiol Pol.

[3] Sterliński M, Oręziak A, Przybylski A, Średniawa B (2019). Experts of the heart rhythm section of the Polish cardiac society: opinion on the use of wearable cardioverter-defibrillators in Poland. Kardiol Pol.

[4] Schulz-Menger J, Collini V, Gröschel J (2025). 2025 ESC guidelines for the management of myocarditis and pericarditis: developed by the task force for the management of myocarditis and pericarditis of the European Society of Cardiology (ESC) endorsed by the Association for European Paediatric and Congenital Cardiology (AEPC) and the European Association for Cardio-Thoracic Surgery (EACTS). Eur Heart J.

[5] Büchel J, Balestra GM, Schuler A (2025). Ventricular arrhythmia in patients with acute myocarditis: a cohort study. Heart Lung.

[6] Chyży T, Małecka B, Bednarek J (2023). A wearable cardioverter-defibrillator vest as a diagnostic and therapeutic tool after COVID-19. Kardiol Pol.

[7] Pilawa M, Kaźmierczak J, Kiedrowicz RM (2022). An optimized long QT syndrome differentiating protocol: a new indication for wearable cardioverter-defibrillator vests. Kardiol Pol.

[8] Burch AE, D'Souza B, Gimbel JR, Rohrer U, Masuda T, Sears S, Scherr D (2020). Physical activity is reduced prior to ventricular arrhythmias in patients with a wearable cardioverter defibrillator. Clin Cardiol.

[9] Kovacs B, Müller F, Niederseer D, Krasniqi N, Saguner AM, Duru F, Hermann M (2021). Wearable cardioverter-defibrillator-measured step count for the surveillance of physical fitness during cardiac rehabilitation. Sensors (Basel).

[10] Jastrzebski M, Sasaki K, Kukla P, Fijorek K, Stec S, Czarnecka D (2016). The ventricular tachycardia score: a novel approach to electrocardiographic diagnosis of ventricular tachycardia. EP Europace.

[11] Olgin JE, Lee BK, Vittinghoff E (2020). Impact of wearable cardioverter-defibrillator compliance on outcomes in the VEST trial: as-treated and per-protocol analyses. J Cardiovasc Electrophysiol.

[12] Büchel J, Balestra GM, Schuler A (2025). Ventricular arrhythmia in patients with acute myocarditis: a cohort study. Heart Lung.

[13] Narducci ML, Ballacci F, Giordano F, Collini V, Imazio M (2024). Sudden cardiac death after acute myocarditis with arrhythmic presentation: hunting for risk predictors - a systematic review and meta-analysis. Open Heart.

[14] Grbović A, Jurčević R, Božović-Ogarević S (2026). Wide QRS tachycardia in WPW: when antidromic AVRT imitates VT. Clin Case Rep.

[15] Sterliński M, Przybylski A, Grabowski M (2025). Wearable cardioverter-defibrillator use in clinical settings in Poland: nationwide observational cohort study. Kardiol Pol.

[16] Yalin K, Soysal AU, Ikitimur B (2024). Diagnostic accuracy of Apple Watch Series 6 recorded single-lead ECGs for identifying supraventricular tachyarrhythmias: a comparative analysis with invasive electrophysiological study. J Interv Card Electrophysiol.

